# Measurement of synovial tissue volume in knee osteoarthritis using a semiautomated MRI‐based quantitative approach

**DOI:** 10.1002/mrm.27633

**Published:** 2019-02-15

**Authors:** Thomas A. Perry, Andrew Gait, Terence W. O’Neill, Matthew J. Parkes, Richard Hodgson, Michael J. Callaghan, Nigel K. Arden, David T. Felson, Timothy F. Cootes

**Affiliations:** ^1^ Arthritis Research UK Centre for Epidemiology, Faculty of Biology, Medicine and Health, Manchester Academic Health Science Centre The University of Manchester Manchester United Kingdom; ^2^ Centre for Imaging Sciences, Division of Informatics, Imaging and Data Science University of Manchester Manchester United Kingdom; ^3^ School of Computer Science University of Manchester Manchester United Kingdom; ^4^ NIHR Manchester Biomedical Research Centre Manchester University NHS Foundation Trust, Manchester Academic Health Science Centre Manchester United Kingdom; ^5^ Department of Rheumatology Salford Royal NHS Foundation Trust Salford United Kingdom; ^6^ Faculty of Health, Psychology, and Social Care, Department of Health Professions Manchester Metropolitan University Manchester United Kingdom; ^7^ Manchester University NHS Foundation Trust Manchester United Kingdom; ^8^ Nuffield Department of Orthopaedics, Rheumatology and Musculoskeletal Sciences University of Oxford Oxford United Kingdom; ^9^ Medical School University of Sydney Australia; ^10^ Clinical Epidemiology Research and Training Unit Boston University School of Medicine Boston Massachusetts

**Keywords:** osteoarthritis, segmentation, semiautomated, synovial tissue volume

## Abstract

**Purpose:**

Synovitis is common in knee osteoarthritis and is associated with both knee pain and progression of disease. Semiautomated methods have been developed for quantitative assessment of structure in knee osteoarthritis. Our aims were to apply a novel semiautomated assessment method using 3D active appearance modeling for the quantification of synovial tissue volume (STV) and to compare its performance with conventional manual segmentation.

**Methods:**

Thirty‐two sagittal T_1_‐weighted fat‐suppressed contrast‐enhanced MRIs were assessed for STV by a single observer using 1) manual segmentation and 2) a semiautomated approach. We compared the STV analysis using the semiautomated and manual segmentation methods, including the time taken to complete the assessments. We also examined the reliability of STV assessment using the semiautomated method in a subset of 12 patients who had participated in a clinical trial of vitamin D therapy in knee osteoarthritis.

**Results:**

There was no significant difference in STV using the semiautomated quantitative method compared to manual segmentation, mean difference = 207.2 mm^3^ (95% confidence interval −895.2 to 1309.7). The semiautomated method was significantly quicker than manual segmentation (18 vs. 71 min). For the semiautomated method, intraobserver agreement was excellent (intraclass correlation coefficient (3,1) = 0.99) and interobserver agreement was very good (intraclass correlation coefficient (3,1) = 0.83).

**Conclusion:**

We describe the application of a semiautomated method that is accurate, reliable, and quicker than manual segmentation for assessment of STV. The method may help increase efficiency of image assessment in large imaging studies and may also assist investigation of treatment efficacy in knee osteoarthritis.

## 1 INTRODUCTION

Osteoarthritis (OA) is a painful and debilitating disorder of the synovial joints, with the knee being the most frequently involved painful site.^1^ Synovitis is common in symptomatic knee osteoarthritis, occurring in up to 90% of those affected.^2^ MRI is the optimum technique for assessing soft tissue including the synovium. Contrast agents enhance the appearance of the synovium, thereby allowing differentiation from surrounding features and structures such as an effusion. Synovitis assessed on contrast‐enhanced (CE)‐MRI has been shown to correlate with both macroscopic appearance at arthroscopy and histology.^3^ Thickening of the synovium (as synovial tissue volume [STV]) has been associated with cellular infiltration,^3^ suggesting that STV can be used as a marker of synovial inflammation. In observational studies, STV has been linked with both pain and cartilage loss.^4^, ^5^ Further, it has been shown that STV decreases following intra‐articular corticosteroid injection,^6^ suggesting that STV may be used as a primary outcome in clinical trials to determine treatment efficacy.

STV may be assessed on MRI using a semiquantitative approach for which there are a number of scoring systems, including the Whole‐Organ Magnetic Resonance Imaging Score^7^; the Boston‐Leeds Osteoarthritis Knee Score^8^; and most recently, the MRI Osteoarthritis Knee Score.^9^ These indices assess disease severity across the whole knee joint by assigning an ordinal score (0–3) over several anatomical sites. Quantitative approaches may be more sensitive in capturing change; however, these methods can be time‐consuming due to the lengthy manual segmentation process required to assess MRIs on a slice‐by‐slice basis. This limits their utility in clinical research and practice and has driven the development of computer‐assisted quantitative segmentation software tools.

Several approaches are available for the semiautomated assessment of STV, and they have been described previously. Østergaard compared synovial membrane volume and the time to undertake the assessment of volume using a semiautomated approach with preset thresholds and manual segmentation.^10^ Assessments using semiautomated segmentation took less time than the manual approach (10–20 min vs. 45–120 min). Further, the measurement of volume using either technique was significantly correlated, with volumes reported using a 45% enhancement threshold demonstrating the smallest absolute difference compared to manual segmentation.^10^ In a separate study, Fotinos‐Hoyer et al. described a semiautomated segmentation approach that combined the application of a 2D shape mask with targeted thresholding, and compared the performance against manual segmentation for the quantitative assessment of synovitis and showed good levels of agreement.^11^ The 2D mask was built using an “in‐house developed program.”^11^

In 1998, Cootes et al. described a method of modeling shape and appearance that could be used to distort a region of interest when applied to a target image, thereby leading to a better fit of the applied region—a process known as active appearance modeling (AAM).^12^ To the knowledge of the authors, AAM has not been used previously to build a 3D shape model specifically designed for the assessment of STV using CE‐MRI scans and also has not been used in conjunction with semiautomated methods and applied to STV assessment.

We describe a semiautomated approach to quantitative assessment of STV using a 3D shape model. The aims of this study were to apply this method and to determine the accuracy, reliability, and efficiency (assessment time) of the approach compared with conventional manual segmentation in the assessment of MRIs in men and women with symptomatic knee OA.

## 2 METHODS

### 2.1 Subjects

For the training of the active appearance model, we used sagittal T_1_‐weighted (T_1_‐w) fat‐suppressed (FS) CE MRIs obtained during the course of 2 clinical trials of men and women with symptomatic knee OA: 1) a randomized, crossover trial of brace therapy in symptomatic patellofemoral OA (Brace study),^13^ and 2) an open‐label trial of intraarticular steroid therapy for knee OA (Targeting Synovitis in Knee OA [TASK] study).^6^ Comparison of the semiautomated and manual approach methods was assessed using data from Brace. Assessment of reliability of the semiautomated method was assessed using sagittal T_1_‐w FS CE MRIs in a subset of subjects who had participated in an intervention study of vitamin D therapy in knee OA (the Vitamin D Evaluation in Osteoarthritis [VIDEO] study^14^).

### 2.2 MRI acquisition

In Brace, all patients were scanned with a 1.5T Philips Gyroscan ACS‐NT scanner (Philips, Best, Netherlands). Sagittal T_1_‐w postcontrast fat‐suppressed (TR = 500 ms, TE = 17 ms, 384 × 384 matrix, slice gap = 0.3 mm, slice thickness = 3 mm) scans were acquired in all subjects at baseline and follow‐up. The contrast agent used was Dotarem (gadoteric acid, 0.2 mL/kg). Contrast‐enhanced (CE) scans were acquired ~10 min after intravenous injection. During the course of the trial, individuals had 4 MRI scans performed over an 18‐week period, with at least 1 scan performed before and 2 performed after receiving the intervention.

In TASK, patients were scanned using a 3T Philips MRI scanner. Sagittal postcontrast T_1_‐w FS (TR = 550 ms, TE = 20 ms, 320 × 320 matrix, slice gap = 0.3 mm, slice thickness = 3 mm) scans were acquired. Postcontrast scans were acquired using contrast agent Dotarem (gadolinium‐DOTA, 0.2 mL/kg),^15^ with scans acquired approximately 10 min after administration.^15^ Subjects had scans at baseline prior to intervention and again at approximately 2 weeks following intervention, with a third scan acquired in some patients within 6 months.

In VIDEO, patients were scanned using a 1.5T Signa MRI scanner (GE Healthcare, Chicago, IL) and a dedicated phased‐array knee coil.^16^ Sagittal postcontrast T_1_‐w FS (TR range = 720 to 800 ms, TE range = 15.6 to 16.1 ms, acquisition matrix 256 × 160, slice gap = 0.6 mm, slice thickness = 3 mm) scans were acquired. Axial proton density FS (TR range = 3860 to 4500 ms, TE range = 31.4 to 32.2 ms, matrix 256 × 192, slice gap = 0.2 mm, slice thickness = 4 mm) and coronal short tau inversion recovery (TR = 3000 ms, TE range = 46.3 to 55.5 ms, matrix 256 × 192, slice gap = 0.3 mm, slice thickness = 3 mm) sequences were also obtained and were used to guide segmentation on the sagittal sequences. Postcontrast scans were acquired starting 3 min after intravenous injection of gadodiamide (0.2 mL/kg [Omniscan, GE Healthcare]), with the last scan acquired within 11 min of administration.^16^ Imaging was performed in a subset of subjects at baseline and up to yearly intervals over a 3‐year period.

### 2.3 Semiautomated assessment of synovitis

#### 2.3.1 Preprocessing and training of active appearance model

The key to our semiautomated approach is that it uses active appearance modelling (AAM)^12^ to register a 3D mask to a target image. The 3D mask is used to estimate the regions in an image where STV may occur. Such a model requires a suitably annotated training set, in this case a set of sagittal T_1_‐w FS postcontrast MRIs (N = 249) from the main Brace (147 images from 44 patients) and TASK (102 images from 39 patients) studies. Each training image was loosely annotated by manual segmentation for regions containing synovitis across the whole joint; work completed as part of the Brace and TASK studies. After collating the masks, a groupwise nonrigid registration algorithm^17^ was used to estimate the mean shape and appearance of the regions that were defined on the training image set. By doing so, the masks defining the regions overlaying synovitis were warped into the mean reference frame and averaged to create a mean 3D mask. Further, deformation fields mapping each of the training images to the mean mask were created using the nonrigid registration algorithm. The original images and associated deformation fields were used to train the AAM. The model was trained to capture synovitis across the whole joint, including infrapatellar region (Hoffa’s fat pad), suprapatellar region, and region posterior to the posterior cruciate ligament.

#### 2.3.2 Application

**Figure 1:**
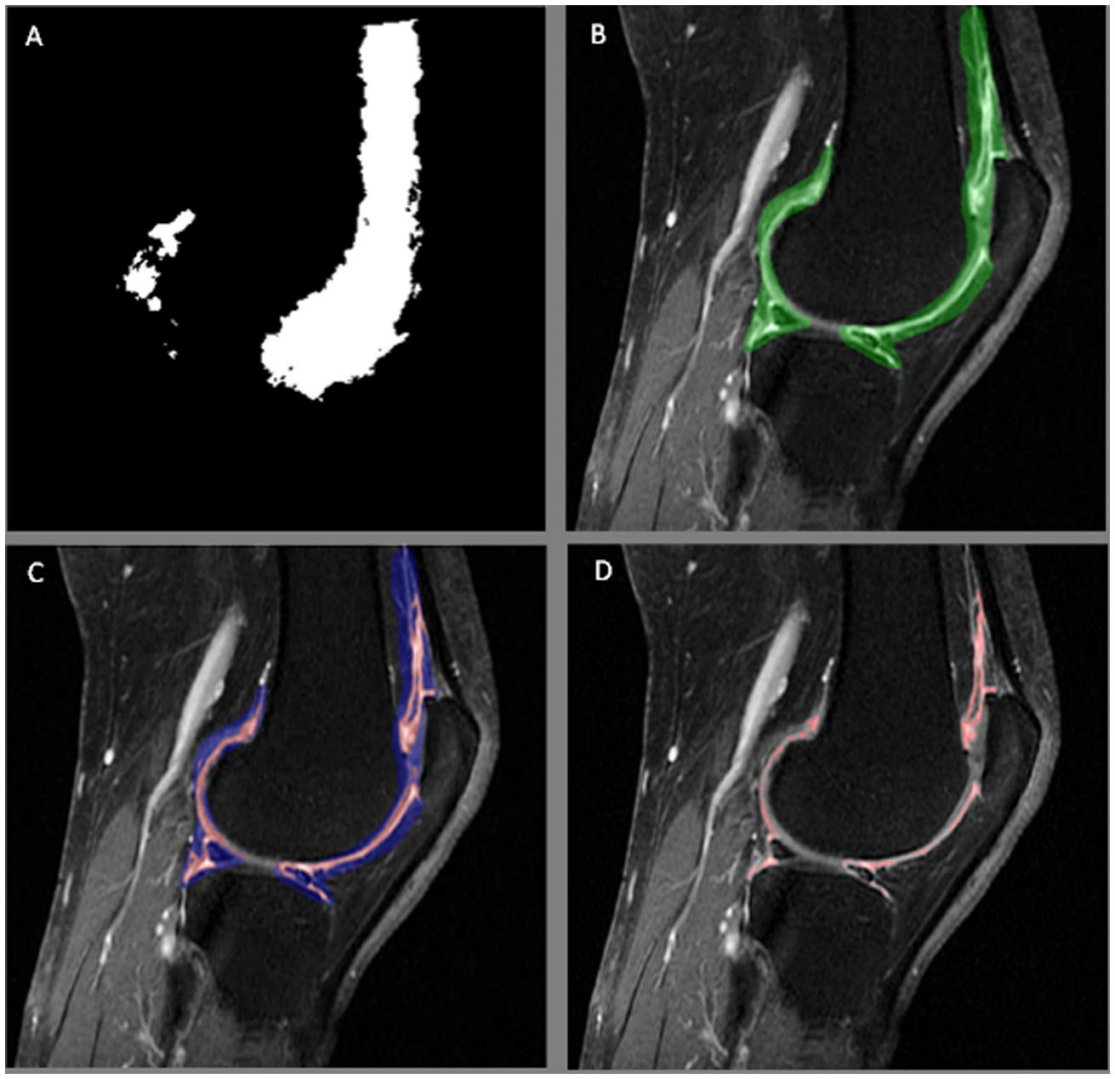
Stages of semi‐automated quantitative assessment of STV using our approach. (A) Preprocessing and development: Mean of masks deformed into a reference frame created for each slice in the MRI sequence to loosely identify regions in which synovitis commonly occurs; a 3D mask (the white regions) for a given slice. (B) Application: Synovitis‐shape model was registered to the target image. Position of the mask was moved, and/or manual editing of the applied shape model was then completed on a slice‐by‐slice basis if failing to overlay the synovium. (C) Targeted thresholding, based on voxel signal intensity, was completed manually to identify STV (red) and efficiently remove the remaining tissue and fluid (blue) from measurement. (D) Editing, post‐thresholding, of the remaining voxels was completed where appropriate by adding and removing voxels manually. Automatic calculation of STV was completed by summation of voxels across all slices, generating an absolute total volume. STV, synovial tissue volume

The semiautomated method combines both automated application of a 3D mask and targeted thresholding, with manual editing of the mask both prior to and after targeted thresholding. To assess synovitis on a new image using our approach, 3 key steps were required: 1) the AAM was applied to estimate regions on an image in which synovitis was likely to occur; 2) targeted thresholding identified synovitis and efficiently excluded voxels that did not correspond to synovitis; and 3) manual editing was conducted throughout (see Figure 1). Volume measurements correspond to voxels that remained after all manual editing had been performed.

The AAM searched the image to estimate the nonrigid mapping from the mean model reference frame to the target image. This mapping was then used to warp the mean synovitis mask onto the target image, defining loosely an *L*‐shaped region of interest. To our knowledge, this is the first time a 3D shape model has been used to quantify STV.

**Figure 2:**
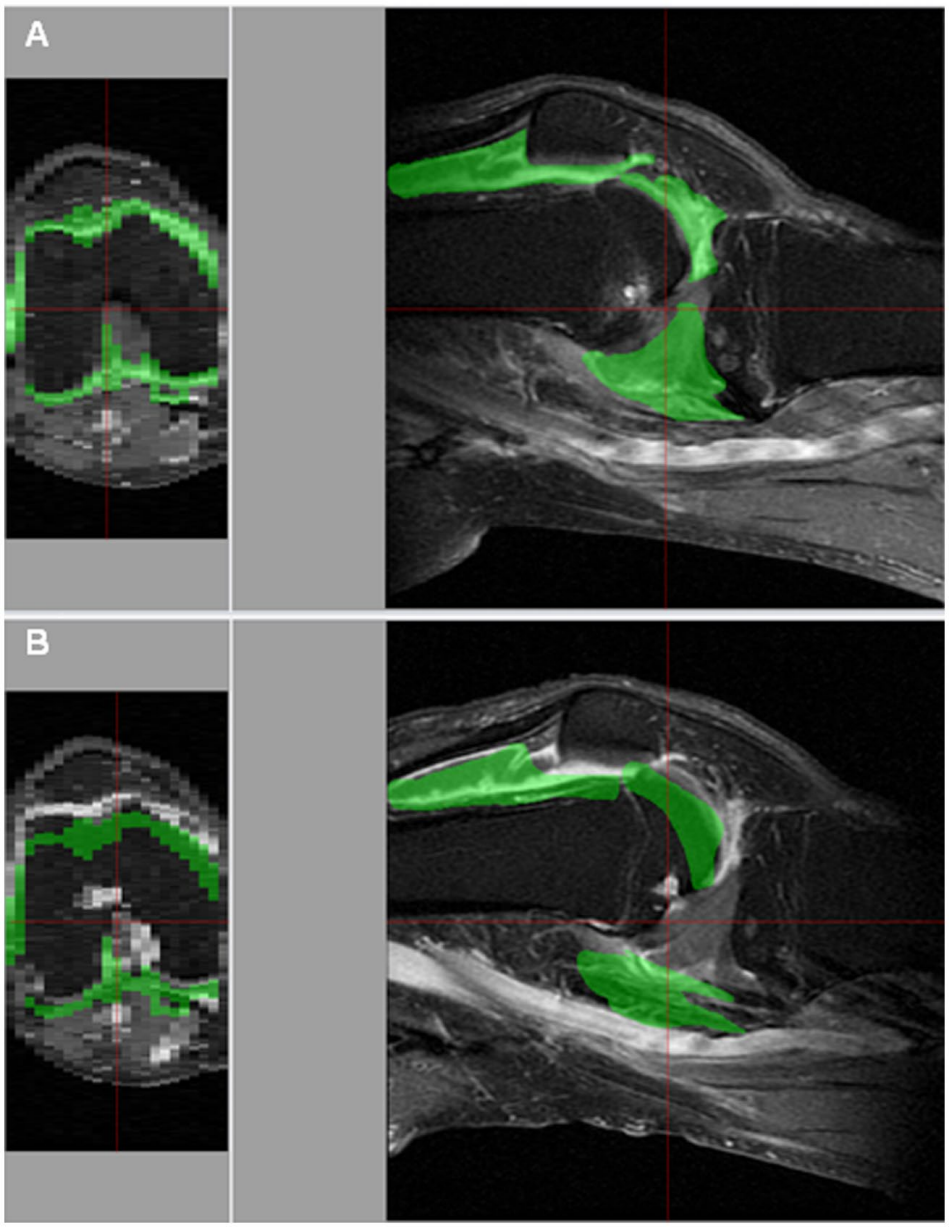
Semi‐automated quantitative assessment of STV using our approach. Software‐computer interface displays a sagittal T_1_‐w postcontrast fat‐suppressed MRI (right) with overlaid synovitis model (green) and a 3D‐rendered axial plane with overlaid synovitis model (left). (A) Correct positioning of synovitis mask to the target image. (B) Displacement in the posterior direction of the synovitis mask to the target image. The displacement of the synovitis‐shape model can also be seen on the 3D rendered axial plane

After registering the mask to the target image, the mask was then assessed by the observer to determine how well it overlaid the synovitis across the whole knee joint. In the event that the mask was poorly mapped to the target image, potentially due to size of the knee or positioning of the knee joint in the FOV (Figure 2B), the mask was manually edited. For instance, the position of the mask could be moved manually, and voxels could be added and/or removed from the mask on the appropriate slice(s) in order to best fit the overlaid synovitis in preparation for thresholding.

**Figure 3:**
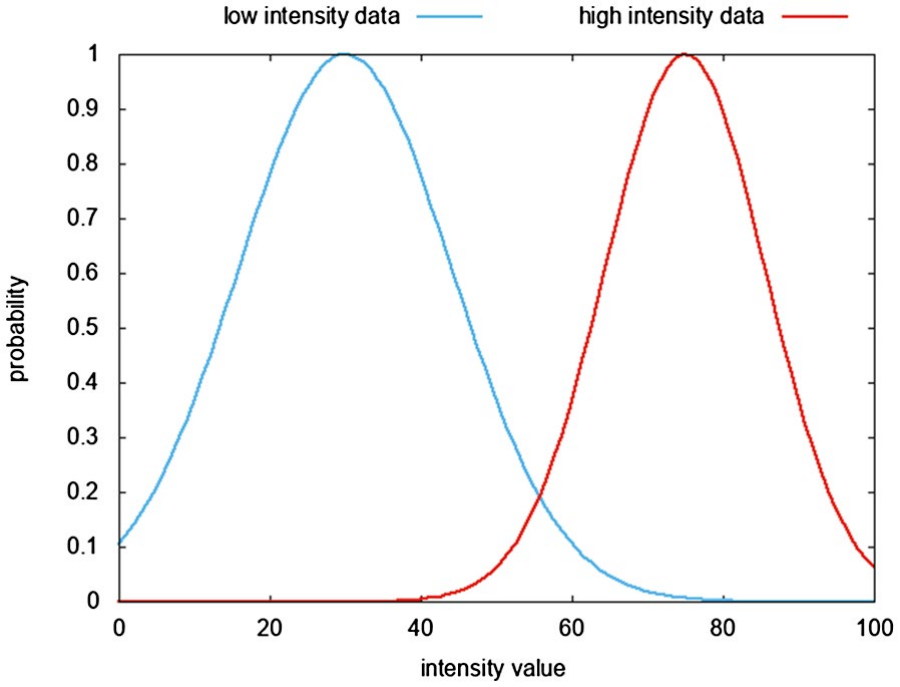
An example of the 2 probability density functions (for low‐ and high‐intensity data) that were used during the thresholding step in the software to determine whether a voxel contained synovitis

Targeted thresholding was then used to identify voxels that were likely to correspond to synovitis and those that were not, and to efficiently remove such voxels from measurement. The software categorized voxels that appeared within the overlaid regions by estimating the proportion of synovitis in each voxel based on 2 thresholds applied across the entire image. Image voxels with intensities below the first threshold (default 25% of the maximum intensity) were grouped into one data set; image voxels with intensities above the second threshold (default 75% of the maximum intensity) were grouped into a second data set. The software assumed that each of these 2 sets of data could be approximated by a Gaussian normal distribution and calculated the probability density functions (PDFs) (see Figure 3). In order to determine whether each voxel within the overlaid mask would be classified as either synovitis or not synovitis, the signal intensity of each voxel would be compared to the 2 PDFs. If the signal intensity of a given voxel had a higher probability in the high‐intensity PDF compared to the probability in the low‐intensity PDF, the voxel was assumed to correspond to synovitis (colored red in Figure 1C); alternatively, if the probability was higher in the low‐intensity PDF, the voxel was assumed to contain no synovitis (colored blue in Figure 1C).

The default values for this thresholding function could be adjusted by the user in the semiautomated segmentation process in order to best capture synovitis and maximize the efficiency of removing voxels that did not correspond to synovitis. Manual segmentation was again employed; however, here it was used to remove any voxels identified that were not synovitis and to add any considered to be synovitis on a per image slice basis. This sequence of thresholding followed by deletion and manual segmentation could be repeated more than once if optimal identification of synovitis could not be achieved solely by using the first thresholding step. Voxels that remained identified following manual segmentation were assumed to contain 100% synovitis. Calculation of volume was completed automatically across all slices.

All semiautomated work was completed on a Dell desktop computer (Intel Core 2 Duo, Dell Inc., Round Rock, TX) running a Microsoft Windows operating system (Microsoft Corp., Redmond, WA). Further, all manual segmentation was performed on a Dell monitor using OsiriX version 8.0. (Pixmeo SARL, Switzerland)^18^ software running on an external Apple operating system (Mac OS X, version 10.6.8; Apple Inc., Cupertino, CA).

### 2.4 Comparison of semiautomated and manual segmentation

To compare the performance of the semiautomated method, 32 sagittal T_1_‐w FS postcontrast images from eight subjects who had taken part in Brace (each participant had 4 MRIs during the course of the study) were manually segmented by a single reader (t.a.p.). The same images were resegmented by the same observer after an interval of 2 weeks using the semiautomated approach. Images were randomized to order, although the segmenter was aware that they came from a single patient. All randomization was performed by a separate member of the research team who took no part in the segmentation procedure. Comparison to manual segmentation is a well‐recognized approach for validating new segmentation methods.^10^, ^11^ Timings of STV measurement were completed by manually timing, in 5‐minute intervals, the duration of the segmentation procedure from the point of mask application through to volume calculation.

### 2.5 Reliability of assessment using the semiautomated method

To assess reliability of the semiautomated method, 12 baseline images from 12 patients who had taken part in the VIDEO study were randomly selected. These were then evaluated using the semiautomated method by a single reader (to.a.p.). The 12 repeats were incorporated into a list of 50 patients. Repeat segmentation of the 12 images occurred over a period of up to 10 weeks by the same reader. Interobserver reliability was assessed by the same 12 images being segmented by a second observer, a radiologist (H. Noorveriandi [h.n .]).

### 2.6 Statistical analysis

Baseline descriptive statistics were calculated using means and SDs for normally distributed variables and using frequencies and proportions for categorical variables. We constructed 95% confidence intervals (CIs) around the mean difference between methods (manual and semiautomated) to assess whether they differed in terms of the average measured STV. Reliability (intraobserver and interobserver) was assessed by constructing Bland‐Altman plots,^19^ allowing qualitative assessment of potentially undesirable patterns in the 2 methods’ data. Appropriate transformations of the data (e.g., natural logarithmic transforms) were performed to see whether any obvious dispersal patterns in the data could be easily corrected for using such transformations. Limits of agreement between the manual segmentation and semiautomated method were calculated to quantify reliability. We also calculated 2‐way mixed‐effects intraclass correlation coefficients (intraclass correlation coefficient 3,1) to quantify reliability. Ordinal linear regressions were used to assess the reliability, with the expectation that the resulting (unstandardized) regression coefficient between the 2 methods or ratings should be as close to 1 as possible, indicating perfect agreement. We used a 2‐sample *t* test to formally test the mean time to undertake assessment and to test the difference in change in STV from baseline to 18 weeks follow‐up, which was measured using manual and semiautomated segmentation methods. All statistical analysis was assessed using Stata MP 13.0/14.0 (StataCorp, College Station, TX).

## 3 RESULTS

### 3.1 Subjects

The mean (SD) age of the 8 Brace subjects was 54.4 years (6.4) and 75% were female. All had knee pain; mean Kellgren and Lawrence score^20^ was 2 (0.63); and mean synovitis volume was 23,219.4 mm^3^ (7013.4). TASK data was used for training the automated dataset only and did not feature in the analysis.

### 3.2 Comparison between manual and automated method of assessment

**Figure 4:**
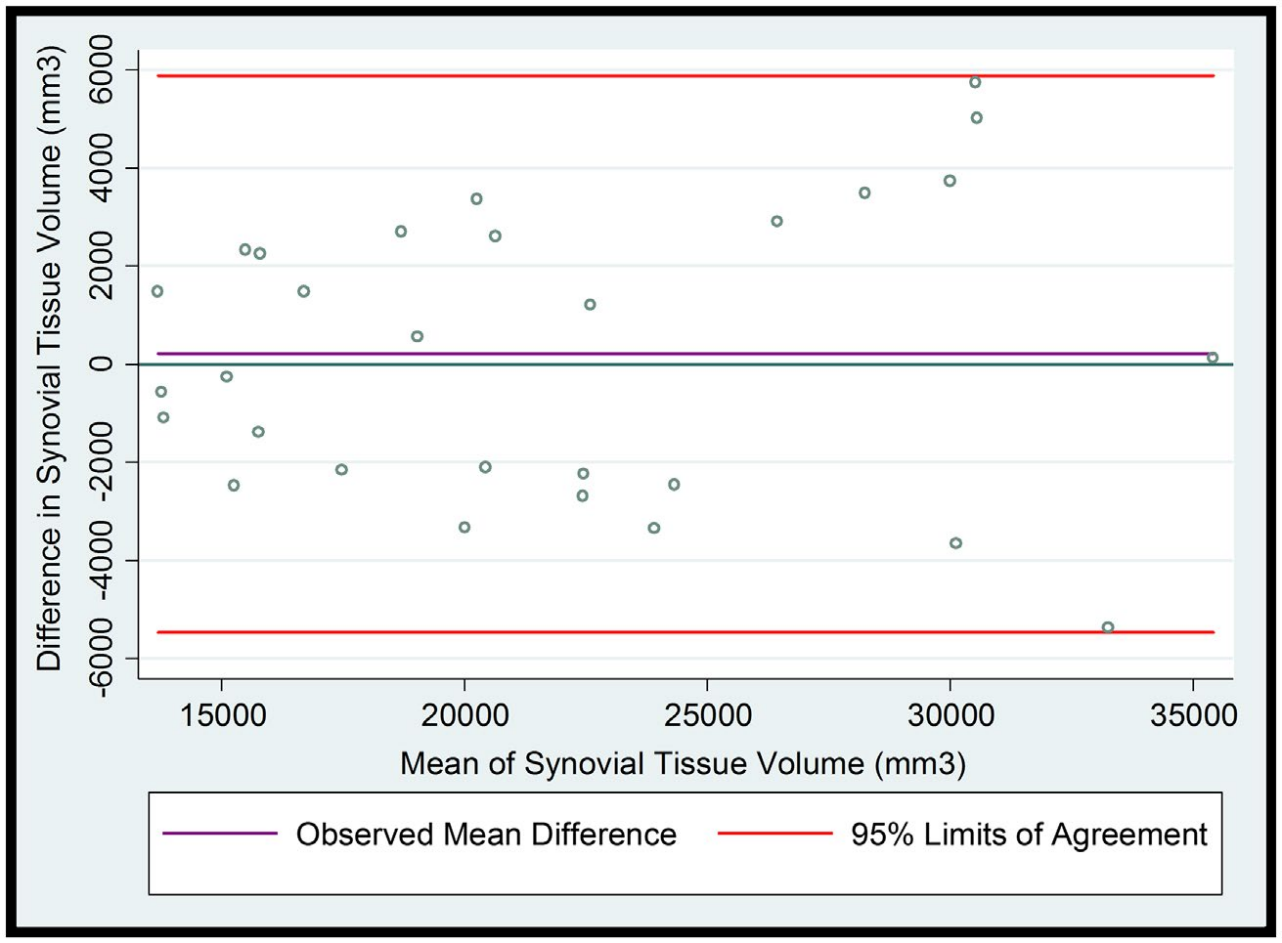
Bland‐Altman plot of differences between semi‐automated segmentation and manual segmentation for the measurement of STV (mm^3^) versus the averages of the 2 segmentation methods for the assessment of STV (mm^3^) (N = 29). Measurements reported using the 2 methods were completed by a single reader (t.a.p.). Data taken from the Brace study

Of the 32 images assessed, 3 images (from 2 patients) were excluded from the analysis due to poor image quality: baseline (N = 1), 12 weeks (N = 1), and 18 weeks follow‐up (N = 1). The mean values for STV for the manual method were 21,690.9 mm^3^ (SD 6432.2) and for the semiautomated method were 21,898.1 mm^3^ (SD 6686.2). Bland‐Altman 95% limits of agreement for measurement of STV were −5473.2 mm^3 ^to 5887.7 mm^3^. The mean difference did not differ significantly from 0 (207.2 mm^3^, 95% CI −895.2 to 1309.7); see Figure 4. As a sensitivity analysis, natural logarithmic transformation was applied to the data, as described by Bland and Altman.^21^ The results were broadly similar, with no statistically significant difference between reported volumes (mean difference: 0.007 log units, 95% CI −0.04 to 0.05). We also tested the difference in mean change in volume from baseline to 18 weeks follow‐up using the 2 approaches. Bland‐Altman 95% limits of agreement for measurement of change in STV were −11,749.0 mm^3 ^to 8917.0 mm^3^. There was no significant difference in mean change in STV measured using the semiautomated approach compared to manual segmentation (−1416.0 mm^3^, 95% CI −6948.7 to 4116.7).

The mean time for manual segmentation of a single image for STV was 71 min (range: 60 to 75), whereas the mean time for semiautomated segmentation was 18 min (range: 15 to 35) using the same data. There was a significant difference in the mean time to undertake assessment of STV using the 2 methods (mean difference = 52.6 min, 95% CI 50.3 to 54.9).

### 3.3 Reliability of semiautomated segmentation

Reliability was assessed by evaluating 12 images from the VIDEO study. In all cases, some alteration was made to the 3D synovitis mask in that voxels were added and/or removed from the mask on at least one slice per scan. In relation to intraobserver reliability, the absolute difference between the first and second assessment ranged from −1087.1 mm^3^ to 959.5 mm^3^, with a mean difference 167.7 mm^3^ (95% CI −199.5 to 534.8). Linear regression between the first and second ratings of the semiautomated approach yielded a slope of 0.99, with an *R* ^2^ of 0.99 for intraobserver agreement. Interobserver agreement was assessed by comparing the results of assessment of 12 images with results obtained independently by a radiologist (h.n.). The absolute difference between reader 1 (t.a.p.) and reader 2 (h.n.) ranged from −5521.9 mm^3^ to 7602.3 mm^3^; mean difference = 1168.7 mm^3 ^(95% CI −1250.1 to 3587.4). The intraobserver agreement was excellent for assessment of STV using the semiautomated method (intraclass correlation coefficient 3,1 = 0.99, 95% CI 0.98 to 0.99), and interobserver agreement was very good (intraclass correlation coefficient 3,1 = 0.83 [95% CI 0.58 to 0.94]).

## 4 DISCUSSION

We describe a semiautomated method of assessment for STV. This is the first study to use active appearance modeling to create the 3D mask that is applied to an image to identify regions in which synovitis is likely to occur. This mask is used to quantify STV. Using the approach described here, STV (mm^3^) assessed produced quantitative volumes that were comparable to manual segmentation and was more efficient in terms of time taken for image assessment. Further, our approach was reliable in the assessment of STV using CE‐MRI.

Manual segmentation is considered to be an accurate and reliable method for the assessment of synovitis on CE‐MRI, and effusion‐synovitis on noncontrast enhanced MRI, with intraobserver agreement up to 0.94^6^ and 0.97.^22^ Manual segmentation is, however, time‐consuming for the measurement of synovitis and can take between 45 to 120 min per case.^10^, ^11^ The timings of STV assessment using our approach are comparable to previous studies that have examined the use of semiautomated segmentation software in knee OA. Fotinos‐Hoyer et al. describe a method of STV assessment in which a 2D method was combined with targeted thresholding using sagittal CE‐MRI.^11^ In their methodology, a 2D oval‐shaped mask that was nonspecific to STV was applied through the MRI stack. Following application and adjustment to the position of the oval mask (if required), targeted thresholding was performed. The total process time using semiautomated segmentation was between 10 to 15 min per knee, which was slightly quicker than our method (mean: 18 min). A longer processing time using our method was to be expected due to the inclusion of manual editing of voxels both prior to and following targeted thresholding. In the Fotinos‐Hoyer study, intraobserver agreement of semiautomated segmentation was tested on 5 knees with repeat assessment after 2 weeks. Linear regression modeling of the repeats yielded a slope of 0.95 and *R* ^2 ^of 0.92.^11^ This compares with our data in which our semiautomated approach had a regression coefficient of 0.99 and *R* ^2^ was 0.99 for intraobserver agreement.

Østergaard^10^ compared semiautomated segmentation to manual segmentation for the measurement of synovial membrane volume. The semiautomated approach, which combined the manual contouring of regions of interest on each 2D axial slice with the application of preset threshold values, required 10 to 20 min per knee.^10^ Timings of assessment are comparable to our approach. Intraobserver agreement using semiautomated segmentation with 5 different threshold values was tested on 3 knees, with repeat assessment performed by a single reader after 2 to 5 days.^10^ The absolute differences for repeat assessment, using the 45% signal intensity threshold that correlated strongest with manual segmentation, were 1, 4, and 12 mL, respectively.^10^ For our study, we performed repeat assessment on a large sample of 12 images, and the differences between the first and second reading as performed by the same reader using semi‐automated segmentation ranged from −1.09 to 0.96 mL.

Our semiautomated approach has a number of benefits over existing tools. Firstly, our synovitis‐shape model is 3D, whereas previous methods have used 2D masks.^11^ A mask was generated for each slice in the MRI sequence, which is more targeted than the approach described by Fotinos‐Hoyer et al.^11^ Further, using our approach and an active appearance model, the mask is automatically adjusted to each target knee. Manual editing was an integral part of this approach, and its inclusion can be viewed as an advantage. For instance, blood vessels enhance on CE‐MRI and often express voxel intensity comparable to synovitis. It was felt that by manually adjusting for these false positives, capture of true synovitis would be achieved.

There are several limitations to be considered in interpreting the data. When comparing the 2 study methods, the images were read in order with the OsiriX images evaluated first and then images assessed using semiautomated segmentation. It is possible that recall of the image when evaluating using OsiriX may have influenced markup when using the semiautomated tool, potentially influencing the findings. However, there was a time delay between assessments of at least 2 weeks, which would tend to mitigate against any recall bias. Ideally, the single reader should have been blinded to the 2 software tools, but this is not practically possible because both processes have individual user interface requirements that subsequently identify the software tools when performing image analyses. The mask was generated using data from 2 separate studies, whereas reliability was assessed in a separate study to those used for AAM development. In the reliability study, the images were acquired using a different MRI protocol, and in all cases the mask required manual adjustment to overlay the STV on the assessed images. Ideally, the mask would be generated from data from the study for which its use was intended; however, the good reliability data described here suggests robustness with the approach. A potential limitation was that an identical MRI protocol was not used across the 3 studies.

A further limitation is that the comparative study was performed using a subsample that was used to generate and train the synovitis mask. Subsequently, it is possible that the timings of assessment were faster than expected because the model will estimate the deformation fields more accurately than for completely different images, leading to more accurate estimation of the regions where synovitis may occur. It is therefore possible that little adjustment would have been required to adjust the mask to overlay the synovitis. In all cases, the mask mapped well to the target images in identifying regions likely to contain synovitis, although manual editing (i.e., adding/removing voxels) was required on at least a single slice per image in all cases to best capture synovitis. The next stage would be to repeat the comparison using a separate study cohort. In addition, the timings of assessment only reflect the time taken to complete assessment once a 3D mask had been generated. An extensive period of time was required to manually segment the training set on which the model was trained. It is likely that the accuracy of the mask was improved by the large data set, and subsequently the large numbers of masks used to train the model.

An additional limitation is the study sample size. For this study, given the time‐consuming nature of the segmentation process, we were limited to using a small subsection of the Brace and VIDEO trials. Ideally, the full trial dataset would be used to give the best estimates of reliability; however, the time taken to do this would be prohibitive, and the sample sizes used were comparable to typical imaging reliability studies.

## 5 CONCLUSION

We describe the application of a semiautomated method of assessment of STV, which is accurate, reliable, and quicker than manual segmentation. The method offers an approach to more rapid assessment of images from large scale observational data or clinical trials.
